# A Case Report of Takayasu's Arteritis and Ulcerative Colitis in a Pediatric Patient with Chronic Recurrent Multifocal Osteomyelitis Successfully Treated with Infliximab: Diagnostic Clues in Disease Associations and Immune Dysregulation

**DOI:** 10.1155/2019/8157969

**Published:** 2019-06-11

**Authors:** Viveka Clare De Guerra, Humaira Hashmi, Bree Kramer, Rula Balluz, Mary Beth Son, Deborah Stein, Alicia Lieberman, Mahmoud Zahra, Rabheh Abdul-Aziz

**Affiliations:** ^1^Department of Pediatrics, University at Buffalo, Oishei Children's Hospital, 1001 Main Street, Buffalo, NY 14203, USA; ^2^Western New York Pediatric Gastroenterology, 166 Washington Avenue, Batavia, NY 14020, USA; ^3^Department of Pediatric Critical Care, University at Buffalo, Oishei Children's Hospital, 1001 Main Street, Buffalo, NY 14203, USA; ^4^Department of Pediatric Cardiology, University at Buffalo, Oishei Children's Hospital, 1001 Main Street, Buffalo, NY 14203, USA; ^5^Department of Pediatric Rheumatology, Boston Children's Hospital, 300 Longwood Ave, Boston, MA 02115, USA; ^6^Department of Pediatric Nephrology, Center for Midaortic Syndrome and Renovascular Hypertension, Boston Children's Hospital, 300 Longwood Ave, Boston, MA 02115, USA; ^7^Department of Pediatric Rheumatology, Strong Memorial Hospital at the University of Rochester, 601 Elmwood Ave, Rochester, NY 14642, USA; ^8^Department of Diagnostic and Interventional Radiology, University at Buffalo, Oishei Children's Hospital, 1001 Main Street, Buffalo, NY 14203, USA; ^9^Department of Pediatric Rheumatology, University at Buffalo, Oishei Children's Hospital, 1001 Main Street, Buffalo, NY 14203, USA

## Abstract

**Background:**

Takayasu's arteritis with comorbid chronic recurrent multifocal osteomyelitis and ulcerative colitis is rare in the pediatric population. Treatment with anti-TNF alpha agents such as infliximab has been a successful treatment strategy in adults and can be used effectively in the pediatric population.

**Case Presentation:**

We present the case of a 15-year-old Caucasian girl with a history of chronic recurrent multifocal osteomyelitis and ulcerative colitis presenting with hypertensive emergency secondary to Takayasu's arteritis with middle aortic syndrome. She was treated with corticosteroids and methotrexate and ultimately required infliximab infusions of 15 mg/kg every 4 weeks to successfully control her symptoms and normalize her inflammatory markers.

**Conclusions:**

In this case, we discuss the use of infliximab in an adolescent patient with chronic recurrent multifocal osteomyelitis, ulcerative colitis, and Takayasu's arteritis. The significance of this case is determined by the unique occurrence of all three conditions in a pediatric patient, the important consideration of vasculitis in the differential of a pediatric patient presenting with hypertensive emergency, the need for vigilance for detecting diagnostic clues, signs, and symptoms, knowledge of disease associations when evaluating a patient with a predisposition for autoinflammatory conditions, and the use of increasing doses of infliximab to control symptoms.

## 1. Introduction

Chronic recurrent multifocal osteomyelitis (CRMO) is a noninfectious, autoinflammatory disorder resulting in repetitive sterile inflammatory lesions in bones [1]. CRMO has been described in association with other inflammatory conditions occurring in childhood including ulcerative colitis [2] and Takayasu's arteritis [3].

Takayasu's arteritis (TA) is an idiopathic large-vessel vasculitis that affects mainly the aorta and its branches [4]. Granulomatous inflammation results in arterial stenosis, thrombus, and aneurysm formation [4]. Proinflammatory cytokines such as TNF alpha play a role in the pathogenesis of TA, and anti-TNF alpha drugs have been utilized as an effective therapy [5]. The Pediatric Rheumatology European Society (PRES) published the current pediatric classification criteria for TA with endorsement by the European League against Rheumatism (EULAR) [6, 7]. Classification criteria requires the presence of angiographic abnormalities of the aorta or its main branches and/or pulmonary arteries (aneurysm, dilatation, narrowing, occlusion, or arterial wall thickening not due to fibromuscular dysplasia) along with at least one of the following five features: pulse deficit, systolic blood pressure difference >10 mmHg between any limb, bruits, or thrills over the aorta and/or its major branches, hypertension, and elevated acute-phase reactant [6, 7]. Current treatment options for TA in children include corticosteroids, cyclophosphamide, methotrexate, and biologic therapies such as TNF-alpha blocking agents [4]. Several adult trials have shown that anti-TNF agents can be successful in treating refractory or therapy-resistant TA [4]. Hoffman et al. demonstrated that 14 of 15 patients with active and relapsing TA showed improvement after the addition of anti-TNF therapy [8]. Fourteen out of fifteen patients with refractory TA also responded well to infliximab 3–5 mg/kg infusions in a multicenter trial by the French Vasculitis Study Group [9].

Ulcerative colitis (UC) is an inflammatory bowel disease that involves mucosal inflammation of the rectum and proximal colon [10]. Current therapies for pediatric UC include corticosteroids, 5-aminosalicylates (5 ASAs), calcineurin inhibitors, thiopurine immunomodulators, and anti-TNF-alpha medications [11]. Hyams et al. demonstrated that infliximab was successful for induction and maintenance therapy in children with moderate to severe UC who did not respond to corticosteroids in a large multicenter, cohort study [12].

## 2. Case Presentation

**Figure 1:**
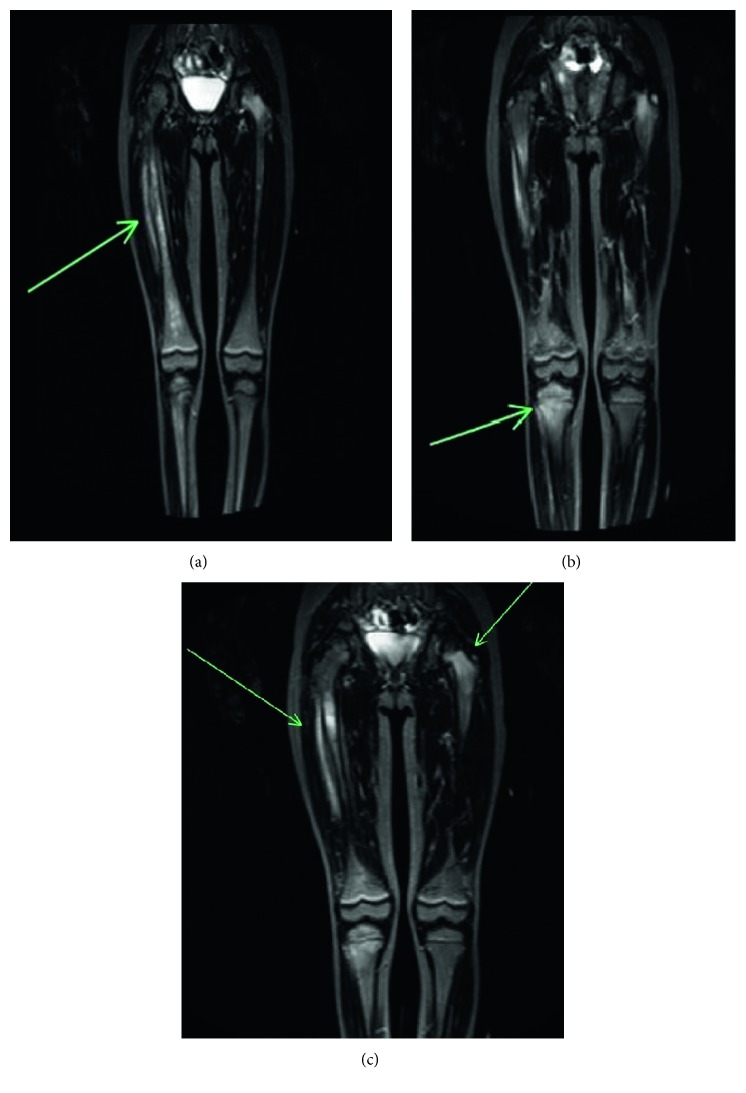
MRI lower extremities showed multifocal abnormal bone marrow signal in the right femur, the left femoral neck, and the proximal epiphysis and metaphysis of the right tibia.

The patient is a 15-year-old Caucasian female who was diagnosed with CRMO in 2007 at 5 years in the context of right thigh pain.  Table 1 provides a timeline of the patient's symptoms and course of disease. Magnetic resonance imaging (MRI) showed multifocal abnormal bone marrow signal in the right femur, the left femoral neck, and the proximal epiphysis and metaphysis of the right tibia, which was associated with osteolysis and callus formation (Figure 1). Radio-nucleotide bone scan found increased uptake involving the left sacrum, left proximal femur, and femoral neck region as well as the midshaft of the right femur and the proximal right tibia. Bone biopsy of the lytic lesion involving the midshaft of the right femur was not consistent with malignancy and showed red blood cells and scattered neutrophils and lymphocytes. Over the next several years (2007–2015), she was followed by orthopedic surgery and was treated only with intermittent ibuprofen as needed for pain. She was noted to have a leg length discrepancy at the age of 7 years, and in 2014, at the age of 12 years, she had surgery to fuse the growth plate to prevent right leg growth (right leg was 4.5 cm longer than her left leg at that time).

She established Rheumatologic care in March 2015 at age 13, and right quadriceps muscle atrophy and failure to thrive with a weight and height under the third percentile were noted at this time. Bone scan revealed increased uptake in the right femur and asymmetry of activity in the growth plates of the knees and ankles with decreased activity in the right side compared to left. She was treated with naproxen 250 mg twice daily (8.7 mg/kg BID) and prednisone 20 mg per day (0.7 mg/kg), tapered by 5 mg weekly. Two months later, prednisone was discontinued, and she continued to have good control of her leg pain on NSAID monotherapy. After a few months, she developed new diarrhea with vomiting and weight loss. Her blood pressure was elevated, and a renal ultrasound and electrocardiogram were unremarkable.

**Figure 2:**
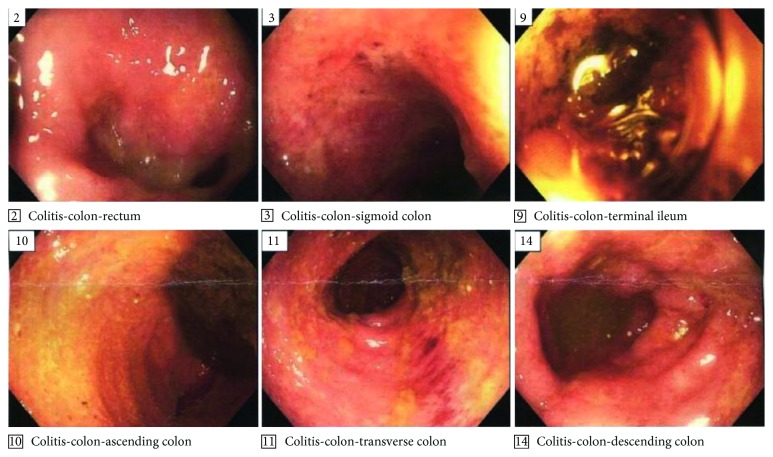
Colonoscopy showed pancolitis with crypt inflammation and crypt abscesses.

She was referred for Gastroenterology evaluation and was found to have positive stool occult blood along with a perirectal skin tag. Laboratory studies revealed anemia with a hemoglobin of 7.1 g/dL (nl 12–16 g/dL), thrombocytosis with platelets of 744 k (nl 150–450 k), erythrocyte sedimentation rate (ESR) of 69 mm/hr (nl 0–10 mm/hr), and C-reactive protein (CRP) of 129 mg/L (nl 0–3 mg/L). There was no family history of autoimmune disease, and tuberculin testing was negative. Colonoscopy revealed pancolitis with crypt inflammation and crypt abscesses with no granuloma consistent with ulcerative colitis (Figure 2). NSAID therapy was discontinued, and treatment for UC with prednisone 1 mg/kg/day (40 mg) tapering by 5 mg weekly along with sulfasalazine was initiated. Infliximab 3 mg/kg infusions were added the following month.

**Figure 3:**
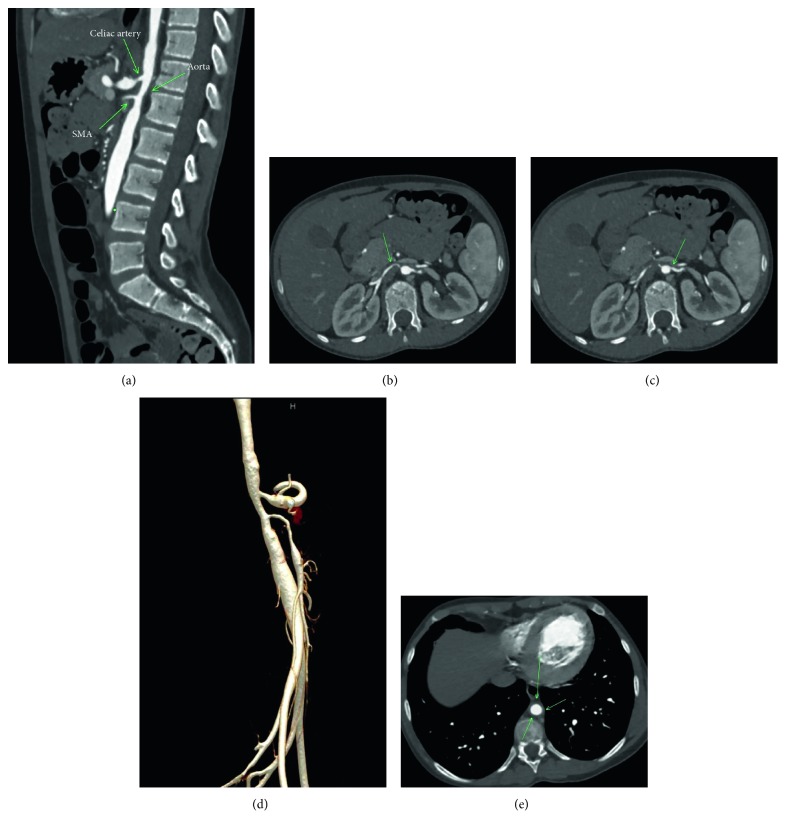
(a) CTA abdomen and pelvis showed narrowing of the mid-aorta, proximal renal arteries, celiac artery, and superior mesenteric artery; (b) CTA abdomen and pelvis showed narrowing of the mid-aorta and right proximal renal artery; (c) CTA abdomen and pelvis showed narrowing of the mid-aorta and left proximal renal artery; (d) CTA abdomen and pelvis showed narrowing of the mid-aorta, proximal renal arteries, celiac artery, and superior mesenteric artery; (e) CTA abdomen and pelvis showed narrowing of the mid-aorta.

**Figure 4:**
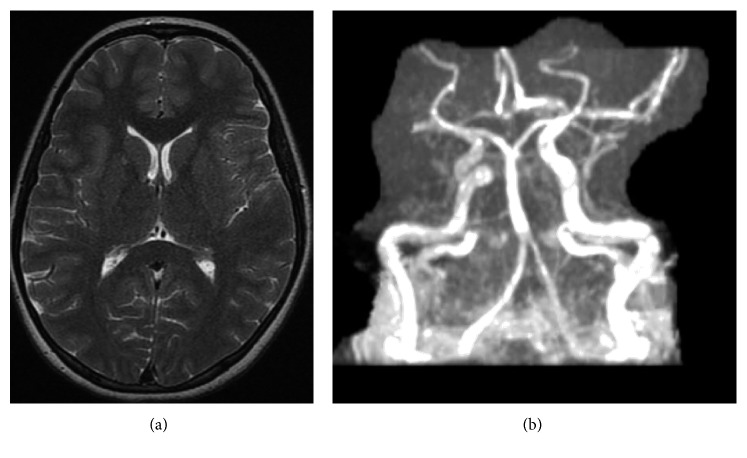
(a) MRI brain within normal limits; (b) MRA brain within normal limits.

At the visit for her second infliximab infusion, she presented with tachycardia, a blood pressure of 230/190, and headache and was admitted to the pediatric intensive care unit for hypertensive emergency requiring nicardipine infusion. Her examination was notable for right-sided Horner's syndrome, decreased right leg pulse pressure, and an abdominal bruit. Echocardiogram showed a small pericardial effusion with reduced left ventricular ejection fraction. Laboratory studies revealed negative ANA and ANCA screens, normal C3 and C4, and normal von Willebrand factor antigen. CT angiogram (CTA) of the abdomen and pelvis showed narrowing of the mid-aorta, proximal renal arteries, celiac artery, and superior mesenteric artery (Figures 3(a)–3(e)). CTA of the chest showed marked descending thoracic and abdominal aortic wall thickening with progressive luminal narrowing and wall thickening of the visualized portion of the right common carotid artery and celiac trunk, enlarged left atrium, left ventricular hypertrophy, and a small pericardial effusion (Figure 3(e)). MRI/MRA brain showed anterior and posterior circulations of the brain were without occlusion or aneurysm with the patent carotid and vertebral arteries of the neck (Figures 4(a) and 4(b)). Given these findings, she was diagnosed with TA complicated by middle aortic syndrome. She was started on metoprolol 50 mg daily (1.5 mg/kg daily), amlodipine 5 mg daily, famotidine 20 mg twice daily, aspirin 81 mg daily, and increased dose and frequency of infliximab from 3 mg/kg every 8 weeks to 5 mg/kg IV every 4 weeks along with prednisone 20 mg daily (0.6 mg/kg daily) and mesalamine 1 g BID. The patient continued infliximab every 8 weeks rather than every 4 weeks as recommended.

Magnetic resonance (MR) angiography of the chest, abdomen, and pelvis and Cardiac MR three months later showed luminal narrowing of the distal thoracic and upper abdominal aorta similar to previous CT studies, stenosis of origin of celiac axis, stenosis of proximal superior mesenteric artery (SMA), and moderate stenosis of bilateral proximal renal arteries. Echocardiogram noted concentric LVH with mildly reduced function, measuring 45%, and normal coronary arteries.

At this point, the patient transferred her care to our rheumatology clinic, and despite treatment with infliximab 5 mg/kg every 8 weeks, she continued to report right thigh pain and developed new inflammatory arthritis of the left ankle and increased inflammatory markers with an ESR of 55 mm/hr (nl 0–20 mm/hr) and an elevated CRP of 73 mg/L (nl < 3 mg/L). Additionally, interval imaging found new wall thickening around the right common carotid artery. This was concerning for uncontrolled TA and CRMO activity. There was an unfortunate delay in treatment escalation due to social circumstances. Two months later, she started treatment with parenteral methylprednisolone 1 gram weekly for 8 weeks, and her dose of infliximab was increased from 5 mg/kg to 10 mg/kg every 4 weeks. Based on adult data demonstrating a positive response to higher doses of infliximab, the decision was made to increase the dose of infliximab instead of trying another TNF inhibitor.

Multidisciplinary evaluation at Boston Children's Hospital, Center for Middle Aortic Syndrome by neurosurgery, nephrology, and rheumatology, led to recommendations of a prednisone dose increase and the addition of methotrexate 15 mg/m^2^ weekly to infliximab 10 mg/kg every 4 weeks. At this time, her echocardiogram revealed moderate left ventricle dilation and mildly depressed left ventricular systolic function. One month later, repeat head and neck CTA showed progression of her right carotid artery stenosis to 80%. Her ESR had normalized, and CRP decreased to 7.8 mg/L at this time. Given her worsening carotid artery stenosis, infliximab was increased from 10 to 15 mg/kg every 4 weeks. On a combination of moderate dose prednisone, weekly methotrexate, and infliximab, she denied joint pain, swelling, abdominal pain, diarrhea, or blood in stool. Follow-up brain MRI/MRA three months later was normal. Repeat chest and abdomen MRA showed all of the areas of stenosis appeared to be stable and inflammatory markers had normalized.

Currently, her clinical course is stable without further anatomic progression, and she has normal inflammatory markers. Thus, she has continued on her current regimen with infliximab 15 mg/kg every 4 weeks and methotrexate 15 mg/m^2^ once weekly. Prednisone decreased gradually and discontinued. She has not developed any infectious sequelae on this regimen. Her blood pressure is stable on carvedilol alone. We continue to assess blood work monthly. Given her use of prednisone, vitamin D level was followed and found to be low, and she has started on a vitamin D supplement. Dilated ophthalmologic exam was normal. Repeat MRI of the lower extremities and bone scan show that her CRMO lesions are inactive. Repeat MRI/MRA of the brain, chest, abdomen, and pelvis in June of 2018 show stable changes without the need for stenting. Given the predisposition for autoinflammatory conditions in this patient, the authors are considering genetic testing in search of a monogenic cause that may support a unifying diagnosis.

## 3. Discussion and Conclusions

Takayasu's arteritis and ulcerative colitis share a common pathogenesis. TA is a large-vessel vasculitis characterized by granulomatous inflammation, mediated by inflammatory infiltrates including cytotoxic T cells, macrophages, and natural killer cells. This results in artery intimal proliferation, thickening of the vessel wall, and luminal stenosis [13]. Inflammatory cytokines such as TNF alpha, interleukin-6, and interferon gamma amplify the inflammatory response [13, 14]. Supporting evidence for TNF alpha in the pathogenesis of TA includes its association with granuloma formation [15] and elevated serum TNF alpha and blood mRNA levels in patients with TA [14, 16, 17]. Additionally, TNF alpha has been identified in the vessel wall of large-vessel vasculitis [15]. Likewise, TNF alpha plays a role in the pathogenesis of ulcerative colitis; TNF alpha levels are increased in patients with ulcerative colitis with studies showing that TNF-alpha inhibitors are an effective treatment for ulcerative colitis [18].

CRMO is an autoinflammatory condition that involves sterile inflammatory lesions in bones resulting in bone pain and fever and is frequently associated with inflammation of the gastrointestinal tract and skin. Bazrafshan and Zanjani first described a case report of a 12-year-old girl with CRMO and UC [19]. Ferguson et al. describe an autosomal recessive form of the disease, known as Majeed Syndrome, caused by a mutation in the LPIN2 gene [20]. Likewise, there is a phenotypically similar murine model called cmo that has inflammation of the bone and skin, demonstrating a mutation in the *pstpip2* gene. Notably, the *pstpip2* gene shares sequence homology to the *pstpip1* gene, which is responsible for causing PAPA, an autoinflammatory syndrome characterized by pyoderma gangrenosum, acne, and pyogenic arthritis, thereby demonstrating a possible etiology for disease associations.

Few previous case reports have shown the occurrence of CRMO, UC, and TA. Vettiyil G et al. describe a case report of a 10-year-old girl who developed CRMO, pyoderma gangrenosum, and TA who was treated with prednisolone and mycophenolate mofetil [21]. Prior to this, Dagan et al. reported a similar case, and Ghosn et al. showed an association between TA presenting as malignant pyoderma gangrenosum in a child with relapsing polychondritis [3, 22]. Likewise, Shirai et al. reported the case of a 27-year-old female diagnosed with sclerosing osteomyelitis of the right mandible who developed Takayasu's arteritis eight months later [23]. In our case, the patient developed UC about 8 years after having CRMO. These disease associations suggest an underlying genetic cause for immune dysregulation.

Shared genetic risk factors play a role in pathogenesis of TA and UC [24]. Both conditions have been associated with a common HLA haplotype B52-DR2 [24]. A case report by Chae et al. discusses a HLA-B52-positive 35-year-old Korean male with a 10-year history of ulcerative colitis who presented with pain and swelling of the right neck and was found to have TA [25]. Additionally, Gecse et al. reported a case of a 30-year-old HLA-B52 positive woman with a severe flare of ulcerative colitis and found findings typical of TA on angiography. She was started on infliximab 5 mg/kg infusions with successful remission [26]. These authors suggest the possibility of a common pathogenesis for both UC and TA after an aggressive immune response based on a genetic predisposition for chronic inflammation [26].

Infliximab is a chimeric human-mouse anti-TNF alpha monoclonal antibody that binds to soluble and membrane-bound TNF alpha. It is used for adult and pediatric inflammatory arthritis and inflammatory bowel disease and has been shown to be effective in refractory TA [5]. Its use has been documented in literature in adults with both TA and UC but less commonly in the pediatric population. Stern et al. showed that, in pediatric patients with TA, infliximab was as effective as cyclophosphamide with fewer side effects and thus is a viable alternative to treatment of pediatric TA [27]. Iwańczak et al. has shown that children with moderate to severe ulcerative colitis have achieved remission with infliximab, and it was effective in preventing early colectomy [28]. Eleftheriou et al. completed a retrospective descriptive case series of four children with CRMO or synovitis, acne, pustulosis, hyperostosis, and osteitis (SAPHO) syndrome treated with TNF alpha blockade and found that disease activity was improved for 3 of 4 children at 12 months after starting biologic therapy and the fourth discontinued therapy due to a suspected fungal skin infection [29]. Gudbrandsson et al. concluded that TNF inhibitors appear to inhibit disease progression and improve outcome in TA in an observational study in adults in South Norway from 1999 to 2012 [30]. In addition, anti-TNF alpha was effective in maintaining remission without glucocorticoids in over 60% of adult patients in a study with refractory TA [8], and anti-TNF alpha has maintained remission in these patients with a median follow-up of 28 months [31]. Infliximab has achieved clinical remission in patients with TA refractory to conventional treatment; for instance, Maffei et al. discussed a 47-year-old female with TA who initially failed treatment with 1 mg/kg/day of prednisone and 15 mg/week of methotrexate, resulting in readmission and decreased quality of life, and was started on infliximab with an initial dose 5 mg/kg IV, a second dose was given after 2 weeks, and a third dose was given after 6 weeks and then every 4 to 8 weeks, and after 8 months, she had improved mobility and decreased pain [5].

Given the success with the use of higher doses of infliximab in adult studies in TA, UC, or CRMO, the decision was made to increase the dose of infliximab in our pediatric patient instead of trying another TNF inhibitor such as adalimumab or trying tocilizumab, which would be acceptable alternatives. In the ULTRA 2 trial, adalimumab was more effective than placebo in achieving clinical remission in patients with moderate to severe UC refractive to conventional therapy; however, the improvement was less than seen in the ACT 1 and 2 trials ten years prior with infliximab. However, all the patients in the infliximab trials had never been exposed to anti-TNF alpha agents, while 40% of patients in the adalimumab trial had been exposed to TNF-alpha inhibitors [32]. Adalimumab has also induced remission of anterior scleritis in late TA [33] that may be related to the severity of inflammation. In addition, tocilizumab is another valid option for the treatment of TA as demonstrated by the recent French retrospective multicenter adult study published in July of 2018 with 80% of TA patients achieving remission with steroid sparring results [34]. However, there are limited data discussing the use of tocilizumab for UC and CRMO.

Our case report highlights the use of increasing doses of infliximab to treat UC, TA, and CRMO in a pediatric patient to achieve stable remission. Unfortunately, there was a significant delay in treating this patient, and treatment was escalated only after she started following in our pediatric rheumatology clinic in 2016. One limitation of this case report is that the events prior to 2016 when the patients established her care in our clinic are retrospectively reported by reviewing available medical record and history collected from patient and her family. While there are many promising adult studies, the literature on the use of biologics and dosing in children with both TA and UC is scarce and extremely rare in patients with all three conditions. TNF alpha plays a role in the pathogenesis of TA and UC, and these diseases have been associated with CRMO. The Childhood Arthritis and Rheumatology Research Alliance developed three consensus treatment plans for the first 12 months of therapy for CRMO patients. The three protocols are methotrexate or sulfasalazine, tumor necrosis factor inhibitors with optional methotrexate, and bisphosphonates [35]. Our patient will continue on methotrexate and TNF inhibitor but again unfortunately, that was not started in the first 12 month of her disease. An underlying genetic element to disease susceptibility is suggested in CRMO that may be of key importance to the development of subsequent autoinflammatory disorders and this warrants being vigilant to signs, symptoms, diagnostic clues, and disease associations during evaluation.

This case also raises the possibility that these patients may have a distinct immune milieu stemming from a single genomic etiology that should be elucidated in future studies and supports genetic testing of our patient in the future.

## Figures and Tables

**Table 1:** Timeline of patient's symptoms and course of disease.

Date	Symptoms and exam findings	Laboratory findings and histopathology and procedures	Imaging findings	Therapy administered	Diagnosis
June 2007 (age 5)	Right thigh pain	Bone biopsy of the right femur was not consistent with malignancy and showed red blood cells and scattered neutrophils and lymphocytes	*MRI lower extremities*: multifocal abnormal bone marrow signal in the right femur, left femoral neck, proximal epiphysis, and metaphysis of the right tibia associated with osteolysis and callus formation	Intermittent ibuprofen	CRMO
			*Bone scan*: increased activity involving the left sacrum, left proximal femur and femoral neck, midshaft of the right femur and proximal right tibia		

2007–2015	Leg length discrepancy noted at the age of 7 years	Surgery by orthopedics to fuse the growth plate to stop right leg growth at age of 12 years	*Lower extremity x-ray*: leg length discrepancy, right leg 4.5 cm longer than left leg	Intermittent ibuprofen	

March 2015 (age 13)	Muscle atrophy of the right leg, FTT^1^, weight and height <3^rd^%		*Bone scan*: extensive increased activity in the right femur and asymmetry of activity in the growth plates of the knees and ankles with decreased activity in the right side compared to left	Naproxen 250 mg twice daily (8.7 mg/kg·BID) Prednisone 20 mg per day (0.7 mg/kg) and decreased by 5 mg per week	

May 2015	Good control of her leg pain			Continued naproxen and discontinued prednisone	

February–April 2016 (age 14)	Hypertension BP^2^: 154/84, symmetric pulses, diarrhea, vomiting, abdominal pain, weight loss, and perirectal skin tag	ESR: 69 mm/hr (nl 0–10 mm/hr), CRP 129 mg/L (nl 0–3 mg/L), Hb 7.1 g/dL (nl 12–16 g/dL), platelets 744 k (nl 150–450 k), calprotectin > 2000 *μ*g/g (nl <50 *μ*g/g), positive occult blood	*Renal ultrasound with Doppler*: normal	Sulfasalazine Oral prednisone 20 mg daily (0.6 mg/kg per day) with tapering Infliximab 3 mg/kg every 8 weeks	Ulcerative colitis
		*Colonoscopy* with pancolitis and crypt inflammation and crypt abscesses, with no granuloma	*Electrocardiogram*: unremarkable		

May 2016	Hypertensive emergency with a BP of 230/190 prior to second dose of infliximab, admitted to the PICU, right Horner's syndrome, headache, fatigue, asymmetric pulses, and abdominal bruit	Echocardiogram: LVEF 47% and mild LVH	*CTA abdomen/pelvis*: narrowing of the mid-aorta, proximal renal artery, celiac artery and SMA^4^	Metoprolol 50 mg daily (1.5 mg/kg)^*∗*^ Amlodipine 5 mg daily (0.15 mg/kg) Famotidine 20 mg BID (0.6 mg/kg) Aspirin 81 mg Increase infliximab to 5 mg/kg IV every 4 weeks^*∗∗*^ Increased prednisone to 20 mg daily (0.6 mg/kg) Mesalamine 1000 mg BID (31 mg/kg)	Takayasu's arteritis complicated by middle aortic syndrome
		Negative ANA and ANCA screens and normal C3, C4, and vWbAg^3^	*CTA chest*: marked descending thoracic and abdominal aortic wall thickening with progressive luminal narrowing and wall thickening of the right common carotid artery and celiac trunk. Enlarged left atrium, and left ventricular hypertrophy. Small pericardial effusion		
		ESR 31 mm/hr (nl 0–10 mm/hr) and CRP 23 mg/L (nl 0–3 mg/L)	*MRI/MRA brain*: normal		

August–September 2016 (age 15)	Right leg pain and inflammatory arthritis of the left ankle	ESR 55 mm/hr (nl 0–10 mm/hr) and CRP 73 mg/L (nl 0–3 mg/L)	*MRA chest, abdomen, and pelvis with cardiac MR*: lumen narrowing of the distal thoracic and upper abdominal aorta likely similar to prior CTA. Stenosis of origin of the celiac axis, proximal SMA, and moderate stenosis of bilateral proximal renal artery. Concentric LVH^5^ with mildly reduced function, measuring 45% with the normal coronary artery	Methylprednisolone 1 gram weekly for 8 weeks followed by prednisone taper Infliximab was increased from 5 mg/kg to 10 mg/kg every 4 weeks^*∗∗∗*^	

December 2016	Asymptomatic	ESR 49 mm/hr (nl 0–10 mm/hr) and CRP 78 mg/L (nl 0–3 mg/L)	*Carotid duplex*: minimal stenosis in internal carotid artery bilaterally and wall thickening around the right common carotid artery	Infliximab 10 mg/kg every 4 weeks, Prednisone was increased to 10 mg daily, Methotrexate 20 mg once a week orally (15 mg/m^2^)	
			*Echocardiogram*: moderate left ventricle dilation. Borderline (low normal to mildly depressed left ventricular systolic function)		

January 2017 (age 15)	Asymptomatic	ESR 6 mm/hr (nl 0–10 mm/hr) and CRP 7.8 mg/L (nl 0–3 mg/L)	*Head and neck CTA*: progression of right carotid artery stenosis with about 80% stenosis	Infliximab was increased to 15 mg/kg every 4 weeks Prednisone 10 mg daily Methotrexate 20 mg weekly (15 mg/m^2^)	

June 2017	Denies any complaints	ESR 2 mm/hr (nl 0–10 mm/hr) and CRP 0.2 mg/L (nl 0–3 mg/L)	*MRI/MRA brain*: normal	Infliximab 15 mg/kg every 4 weeks Prednisone 5 mg daily Methotrexate 20 mg weekly (15 mg/m^2^)	
			*MRA chest and abdomen*: stable study as before without worsening or improvement		

June 2018	Denies any complaints	ESR and CRP normal	*MRI/MRA brain*: normal	Infliximab 15 mg/kg every 4 weeks Methotrexate 20 mg weekly (15 mg/m^2^)	
			*MRI/MRA chest, abdomen, and pelvis*: stable changes without the need for stenting		

^1^FTT: failure to thrive; ^2^BP: blood pressure; ^3^vWb Ag: von Willebrand antigen; ^4^SMA: superior mesenteric artery; ^5^LVH: left ventricle hypertrophy; ^*∗*^metoprolol was later changed to carvedilol; ^*∗∗*^patient continued infliximab every 8 weeks rather than every 4 weeks as recommended; ^*∗∗∗*^these were started 2 months later due to social circumstances.

## List of Abbreviations

CRMO: Chronic recurrent multifocal osteomyelitis
TA: Takayasu's arteritis
UC: Ulcerative colitis
LVH: Left ventricular hypertrophy
CTA: Computed tomography angiogram
MR: Magnetic resonance
SAPHO: Syndrome synovitis, acne, pustulosis, hyperostosis, and osteitis.
